# *DhSip1*, an NRPS-independent siderophore biosynthetic gene, regulates adhesive knob formation and pathogenicity in *Dactylellina haptotyla* through mediating iron acquisition

**DOI:** 10.1128/aem.02446-25

**Published:** 2026-03-30

**Authors:** Hong-Mei Lei, Shi-Mei Shen, Ping Xu, Guo-Hong Li, Pei-Ji Zhao

**Affiliations:** 1 State key Laboratory for Conservation and Utilization of Bio-Resources in Yunnan, Yunnan University, School of Life Sciences, Kunming, China; 2 School of Ecology and Environmental Sciences, Yunnan University, Kunming, China; Chalmers tekniska hogskola AB, Gothenburg, Sweden

**Keywords:** *Dactylellina haptotyla*, NRPS-independent siderophore (NIS) biosynthetic gene, iron acquisition, trap formation, pathogenicity

## Abstract

Iron acquisition plays a crucial role in fungal pathogenicity. Nematode-trapping fungi (NTFs) serve as important biocontrol agents that can develop traps to capture and kill nematodes. Here, we characterize a non-ribosomal peptide synthase-independent siderophore biosynthetic gene, *DhSip1*, in NTF *Dactylellina haptotyla*, which modulates trap (adhesive knob) development and pathogenicity by regulating siderophore-dependent iron acquisition. The deletion of *DhSip1* severely impaired adhesive knob formation and reduced nematode mortality. A chrome azurol S assay confirmed that the gene deletion impaired siderophore biosynthesis. Conversely, overexpression of *DhSip1* enhanced these phenotypes. Notably, the pathogenicity defects of Δ*DhSip1* were fully rescued by exogenous iron rescue assay, supporting a functional link between iron acquisition and adhesive knob-mediated infection. Fluorescence localization revealed specific enrichment of *DhSip1* in adhesive knobs during late infection, suggesting that its localization provides a mechanism for local iron enrichment, thereby potentially supporting the formation of adhesive knobs. Collectively, *DhSip1* optimizes predatory efficiency by coordinating iron acquisition with adhesive knob development in *D. haptotyla*. Our work provides new insights into the pathogenicity mechanism of *D. haptotyla* and establishes a theoretical foundation for biocontrol product development.

**IMPORTANCE:**

Fungal pathogens require iron for pathogenicity, but the role of iron acquisition in *Dactylellina haptotyla* remains unclear. This study identifies a novel non-ribosomal peptide synthase-independent siderophore synthetase gene, *DhSip1*, in *D. haptotyla*, which is essential for siderophore production, iron acquisition, and adhesive knob formation. We demonstrate that iron acquisition critically governs both the adhesive knob development and the pathogenicity of *D. haptotyla*. Furthermore, *DhSip1* mediates local iron enrichment within adhesive knobs, revealing a unique pathogenic mechanism that directly links iron homeostasis to nematode predation. Our findings not only advance our understanding of the pathogenic mechanisms in *D. haptotyla* but also pave the way for designing effective biocontrol products.

## INTRODUCTION

Iron is an essential nutrient for the growth, survival, and pathogenicity of microorganisms (1, 2). Many cellular processes, such as energy metabolism, antioxidant stress responses, and DNA synthesis, require iron as a cofactor for catalysis or direct participation (3, 4). To acquire iron, microorganisms produce iron-chelating molecules called siderophores. These small molecules are synthesized under iron-limited conditions, exhibit an extremely high affinity for ferric iron, and are specifically designed to chelate and facilitate the uptake of iron from the environment (5, 6). The biosynthesis of siderophores is mainly catalyzed by two key enzyme systems: non-ribosomal peptide synthases (NRPSs) and non-ribosomal peptide synthase-independent siderophores (NISs) (7). Recent studies indicate that the biosynthesis of siderophores and iron uptake are crucial for fungal pathogenicity. The opportunistic fungus *Aspergillus fumigatus* employs four distinct siderophores for iron acquisition and utilizes ferricrocin (FC) for hyphal iron storage and distribution (8, 9). SidL is a constitutively active *N*^5^-hydroxyornithine-acetylase essential for siderophore biosynthesis. A *sidL* deletion mutant exhibited decreased accumulation of FC-derived hydroxyferricrocin in its conidia, resulting in delayed germination, reduced conidial size, and diminished resistance to oxidative stress (10). *Cryptococcus neoformans* senses iron to regulate the formation of the polysaccharide capsule, which is the major virulence factor during infection (11, 12). *Cir1* is the iron response transcription factor that controls the regulation of genes for iron acquisition. Deleting *Cir1* in *C. neoformans* impairs the ability to sense changes in external iron levels. Consequently, pathogenicity-associated phenotypes, such as capsule formation, production of the antioxidant melanin in the cell wall, and growth at the host body, are all affected (13). A similar relationship between iron and virulence factors (e.g., diphtheria toxin) also exists in bacterial pathogens (14). In agriculture, siderophores can serve as biocontrol agents against pathogens. For example, *Pseudomonas fluorescens* secretes siderophores to acquire iron under low-iron conditions, thereby competitively inhibiting pathogen growth and controlling plant diseases (15, 16). Additionally, siderophore-iron complexes can serve as an iron source for plants, promoting their growth (17, 18).

Root-knot nematode diseases cause a global threat to agricultural production (19–21). Nematode-trapping fungi (NTFs) are important biocontrol materials whose hyphae can specialize into traps to capture nematodes, possessing crucial application value for controlling root-knot nematode populations (22, 23). The pathogenicity of NTF is known to be influenced by various factors, among which siderophore-mediated iron acquisition has recently been identified as a critical factor. For example, in the model NTF *Arthrobotrys oligospora*, the NRPS gene *Ao415* is essential for synthesizing hydroxamate siderophores such as desferriferrichrome; its deletion impairs trap formation and virulence (24). In contrast to the NRPS-dependent genes, the role of the NIS gene in NTF remains largely unexplored. *D. haptotyla* is a highly efficient NTF that employs adhesive knobs to capture nematodes (25). In this fungus, we identified a core gene, *EVM02G002910*, located in a putative NIS gene cluster, which is highly upregulated in response to nematodes (26). Despite this suggestive expression pattern, its specific function in pathogenicity remains unclear.

To elucidate its function, we performed functional characterization of *EVM02G002910* through reverse transcription quantitative PCR (RT-qPCR), gene deletion and overexpression, iron rescue assay, and fluorescent labeling. Given its involvement in siderophore biosynthesis and iron acquisition, the key processes for fungal pathogenicity, we named it *DhSip1*. Our results indicate that *DhSip1* is critical for iron uptake in *D. haptotyla*, and its deletion significantly impairs fungal pathogenicity by inhibiting adhesive knob development. This study provides new insights into the pathogenic mechanisms of NTF.

## RESULTS

### Identification and sequence analysis of *DhSip1*

**Fig 1:**
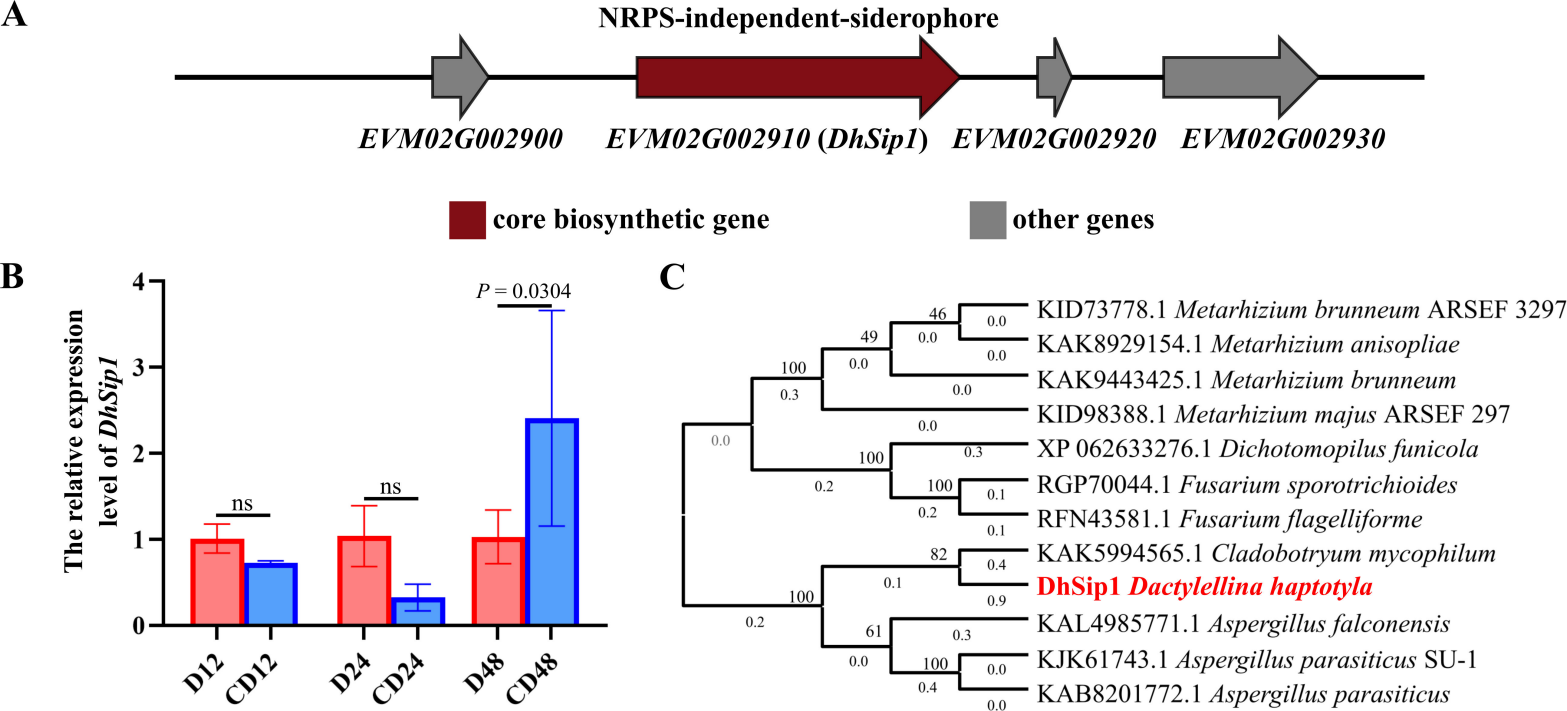
Identification and analysis of *DhSip1*. (**A**) antiSMASH annotation of gene cluster 3.2. (**B**) RT-qPCR analysis of *DhSip1* during *D. haptotyla*’s predation on nematodes. “CD-12,” “CD-24,” and “CD-48” represent the transcript level of *DhSip1* after *D. haptotyla* interacts with *C. elegans* for 12, 24, and 48 h, respectively; “D-1,” “D-24,” and “D-48” represent the transcript expression level of *DhSip1* in control (*D. haptotyla* only). Values are means ± SD. *P* values are shown above the paired columns according to two-way ANOVA; *n* = three biological replicates. (**C**) Phylogenetic analysis of *DhSip1*. MEGA 12 was used to construct the phylogenetic tree, which was built using the maximum likelihood method under the Dayhoff + G + F model of evolution with 100 bootstrap replicates.

In previous works, a significant upregulation of the transcript levels of *DhSip1* was identified during the predation of *D. haptotyla* on nematodes (26). *DhSip1* is the core gene of a NRPS-independent siderophore gene cluster (gene cluster 3.2) with a lucA/lucC domain (Fig. 1A). The cDNA open reading frame of *DhSip1* is 3,168 bp in length (GenBank accession number: PX849498. The corresponding sequence data are publicly available at Figshare: https://doi.org/10.6084/m9.figshare.31769125), encoding a protein of 1,055 amino acids with a predicted isoelectric point of 7.11 and a molecular mass of 119.26 kDa. RT-qPCR analysis confirmed an expression pattern consistent with the transcriptomic data (Fig. 1B), which further suggests that *DhSip1* may be related to the predation. Phylogenetic analysis showed that the protein DhSip1 and KAK5994565.1 (a putative NRPS-independent siderophore synthetase rfs-like protein) from *Cladobotryum mycophilum* are clustered into one clade (Fig. 1C). Notably, the function of KAK5994565.1 remains largely uncharacterized, which indicates that our research focuses on a novel siderophore synthesis gene that had not been characterized previously, offering new insights into their functional diversity.

### *DhSip1* had no significant effect on vegetative mycelial growth

To investigate the role of *DhSip1*, we conducted a gene deletion and overexpression experiment. Targeted gene deletion and overexpression strains were constructed by means of a PEG-mediated homologous recombination (27). Positive transformants*—DhSip1* deletion mutants (Δ*DhSip1*) and *DhSip1* overexpression mutants (*DhSip1* OE)—were confirmed by polymerase chain reaction (PCR) for subsequent experiments (Fig. S1).

**Fig 2:**
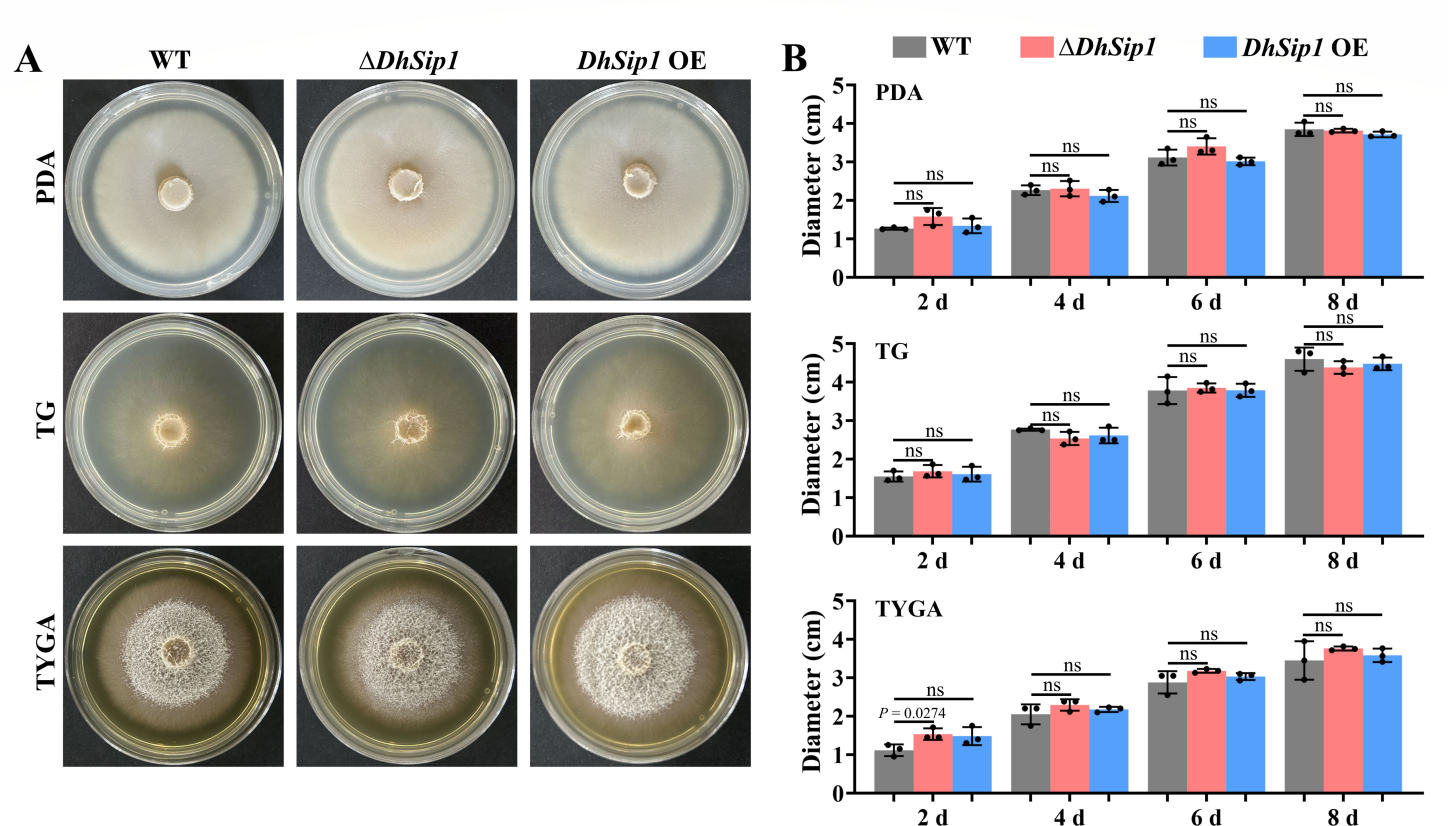
Colony morphology and diameters between the WT and mutant strains (Δ*DhSip1* and *DhSip1* OE). (**A**) Mycelial growth status of WT and mutant strains under different culture conditions. (**B**) Colony diameters of the WT and mutant strains cultured on PDA, TG, and TYGA media at 28°C for 8 days. Values are means ± SD. ns represents *P* values >0.05, which is calculated by unpaired Student’s *t*-test (two tailed); *n* = 3 biological replicates.

The production of siderophores can promote the growth of microorganisms (28). Considering that *DhSip1* is the core gene of a siderophore gene cluster in *D. haptotyla*, we hypothesized that it would play a similar role in mycelial growth. To test this hypothesis, we conducted a comparative analysis of mycelial growth rates. The wild-type (WT) and mutant strains (Δ*DhSip1* and *DhSip1* OE) were cultured on potato dextrose agar (PDA), tryptone glucose (TG), and tryptone yeast extract glucose agar (TYGA) media. The results showed that there was no significant difference in the mycelial growth between the WT and the mutant strains on any of the three media (*P* > 0.05, unpaired Student’s *t*-test, *n* = 3; Fig. 2).

### *DhSip1* was involved in cellular responses to stress cues

To assess whether *DhSip1* affects the response of *D. haptotyla* to different chemical stress, we then performed stress response tests on WT and mutant strains (Δ*DhSip1* and *DhSip1* OE).

**Fig 3:**
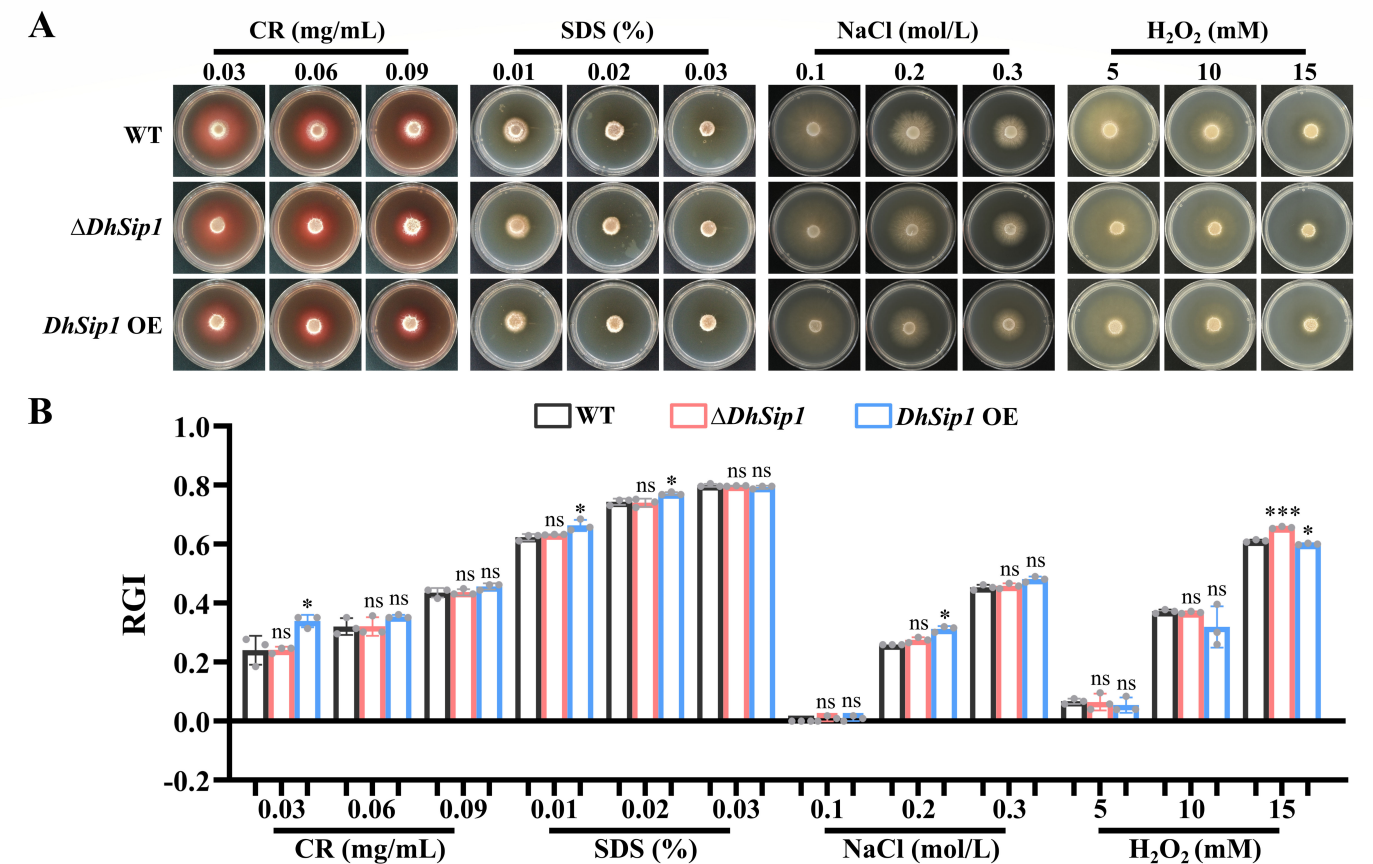
Comparison of stress response to chemical agents between the WT and mutant strains (Δ*DhSip1* and *DhSip1* OE). (**A**) Colony morphology of the WT and mutant strains cultured on TG medium supplemented with different chemical agents. (**B**) Relative growth inhibition (RGI) values of WT and mutant strains under different chemical stressors. Values are means ± SD. ns represents *P* values >0.05; * represents *P* values <0.05; *** represents *P* values <0.001. *P* values represent the mutant strains compared to the WT by unpaired Student’s *t*-test (two tailed); *n* = 3 biological replicates.

On TG medium supplemented with SDS (0.01%, 0.02%, and 0.03%), the growth rate of the Δ*DhSip1* strain was similar to that of the WT, with no significant difference in relative growth inhibition values. In contrast, the *DhSip1* OE strain showed significantly reduced growth rate at 0.01% and 0.02% SDS, with no difference observed at 0.03%. Under Congo red stress (0.03, 0.06, and 0.09 mg/mL), the Δ*DhSip1* strain still showed no significant difference in growth rate compared to the WT, whereas the *DhSip1* OE strain exhibited a significantly lower growth rate than the WT at 0.03 mg/mL Congo red (Fig. 3).

When cultured on TG medium supplemented with NaCl (0.1 and 0.3 mol/L), neither mutant differed from the WT. At 0.2 mol/L NaCl, however, the *DhSip1* OE strain exhibited significantly impaired growth compared to the WT, while the Δ*DhSip1* strain remained unaffected. When treated with 5 and 10 mM H_2_O_2_, the growth rates of the mutant strains were similar to that of the WT. Under 15 mM H_2_O_2_ conditions, the growth rate of the Δ*DhSip1* strain was significantly lower than that of the WT, while the *DhSip1* OE strain showed the opposite phenotype (Fig. 3).

Collectively, these results indicate that *DhSip1* modulates stress tolerance in a condition-dependent manner, with its overexpression often conferring hypersensitivity under certain stressors (e.g., SDS and NaCl) but enhanced resistance under oxidative stress (H_2_O_2_).

### *DhSip1* is required for the pathogenicity

RT-qPCR results showed that the transcript level of *DhSip1* was significantly upregulated during the predation of *D. haptotyla* on nematodes; therefore, we hypothesized that *DhSip1* might play a role in the predation process. To test this hypothesis, we conducted pathogenicity assays and compared the adhesive knob numbers and nematode mortality between the WT and mutant strains (Δ*DhSip1* and *DhSip1* OE).

**Fig 4:**
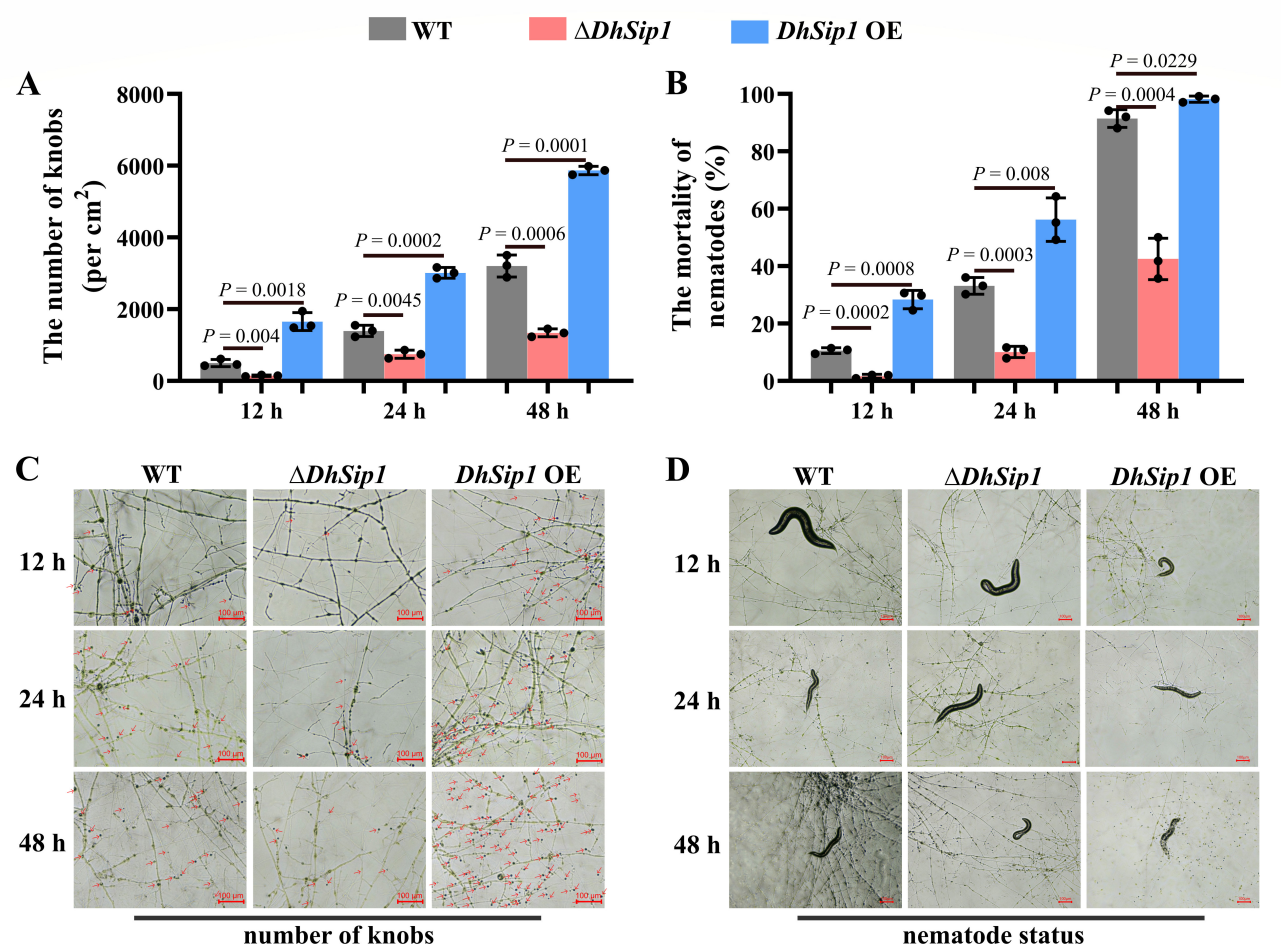
Comparison of the pathogenicity against *C. elegans* between the WT and mutant strains (Δ*DhSip1* and *DhSip1* OE). (**A**) The number of adhesive knobs of WT and mutant strains induced by *C. elegans* at different time points. (**B**) The nematode mortality of WT and mutant strains at different time points. Values are means ± SD. *P* values are shown above the paired columns according to unpaired Student’s *t*-test (two tailed); *n* = 3 biological replicates. (**C and D**) Representative views of WT and mutant strains at different time points after interaction with *C. elegans*.

As the results showed that at the infection time of 12, 24, and 48 h, the average adhesive knob numbers in Δ*DhSip1* were 142, 742, and 1,337/cm^2^, respectively, which were significantly fewer than those in the WT (497, 1,392, and 3,202/cm^2^, respectively) (*P* < 0.01, unpaired Student’s *t*-test, *n* = 3; Fig. 4A and C). Consistent with the reduction in adhesive knobs number, the nematode mortality in Δ*DhSip1* (1.62% at 12 h, 10.08% at 24 h, and 42.50% at 48 h) was significantly lower than that in the WT (10.53% at 12 h, 33.13% at 24 h, and 91.43% at 48 h) (*P* < 0.001, unpaired Student’s *t*-test, *n* = 3; Fig. 4B and D). Conversely, the *DhSip1* OE strain displayed the opposite pathogenicity phenotype. At the infection time of 12, 24, and 48 h, the numbers of adhesive knobs in *DhSip1* OE were 1,652, 3,012, and 5,869/cm^2^, respectively, which were significantly higher than those in the WT (*P* < 0.01, unpaired Student’s *t*-test, *n* = 3; Fig. 4A and C), while the nematode mortality in *DhSip1* OE (28.33% at 12 h, 56.22% at 24 h, and 98.20% at 48 h) was significantly higher than that in the WT (*P* < 0.05, unpaired Student’s *t*-test, *n* = 3; Fig. 4B and D).

Collectively, these results demonstrate that *DhSip1* is essential for the pathogenicity of *D. haptotyla*. The positive correlation between adhesive knob formation ability and nematode mortality led us to hypothesize that *DhSip1* regulates pathogenicity through its role in knob development. To test this hypothesis, we examined the transcription levels of several key trap-development-related genes. Consistent with the pathogenicity observations, all tested genes were significantly downregulated in Δ*DhSip1* (Fig. S2).

### Involvement of *DhSip1* in iron acquisition and pathogenicity

**Fig 5:**
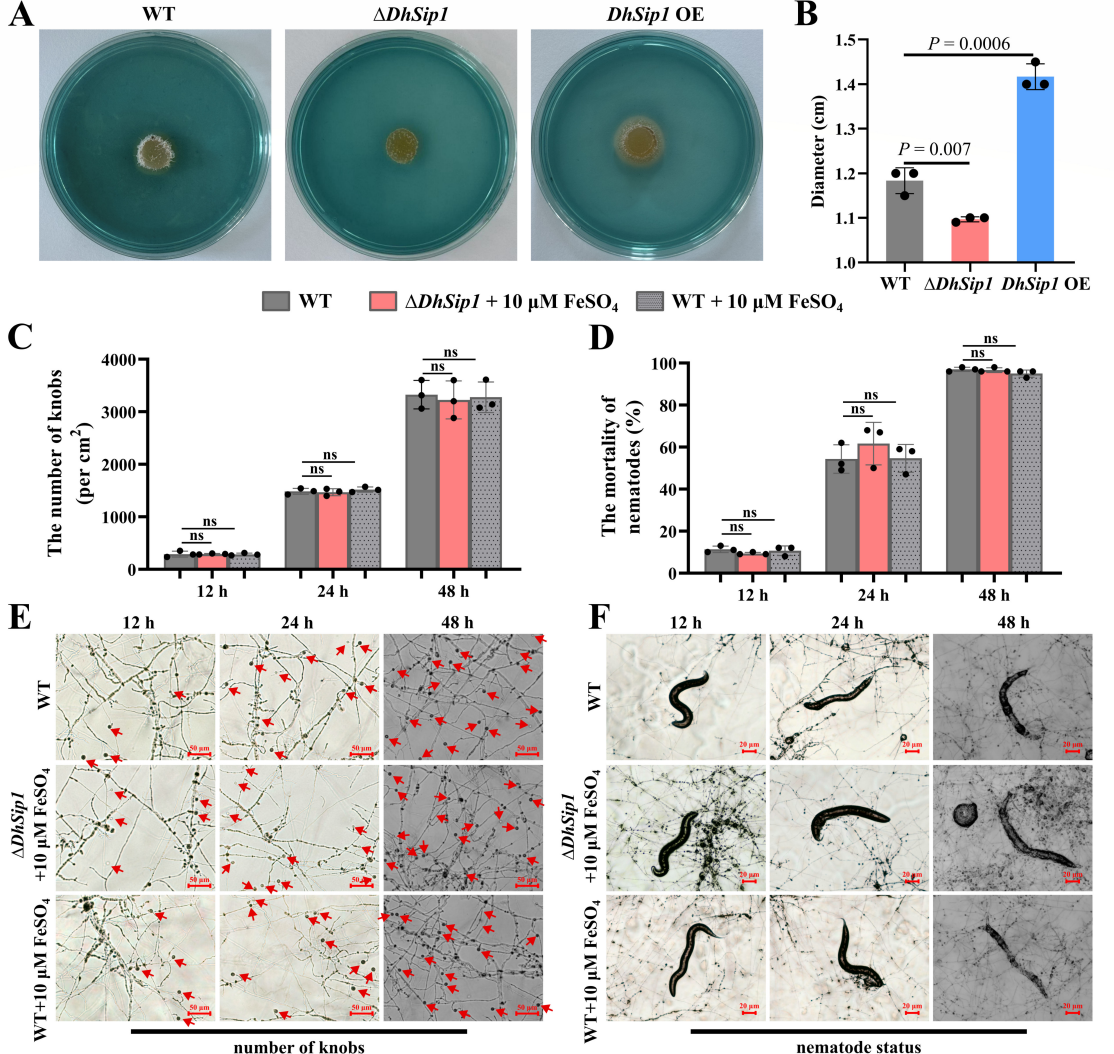
*DhSip1* regulates fungal pathogenicity through iron acquisition. (**A and B**) Siderophore detection in the WT and mutant strains (Δ*DhSip1* and *DhSip1* OE). (**C and D**) Iron rescue assay and its effect on pathogenicity. (**E and F**) Representative views of WT, Δ*DhSip1 +* FeSO_4_, and WT *+* FeSO_4_ control at different time points after interaction with *C. elegans*.

The above results prompted us to hypothesize that *DhSip1* regulates pathogenicity by modulating adhesive knob development. To elucidate the molecular function of *DhSip1* underlying this phenotype, we first assessed its role in siderophore production. Chrome azurol S (CAS) plate assays revealed a distinct pale-yellow halo around the WT and *DhSip1* OE, indicative of siderophore production, with the *DhSip1* OE exhibiting a larger zone. In contrast, the Δ*DhSip1* produced no detectable halo, confirming a severe defect in siderophore synthesis (Fig. 5A and B). Together with its genomic annotation as a siderophore biosynthesis gene, these results establish *DhSip1* as essential for siderophore production in *D. haptotyla*. Given the concurrent impact of *DhSip1* deletion on adhesive knob development, we further speculated that its role in adhesive knob morphogenesis may be functionally linked to siderophore metabolism.

Siderophore metabolism is involved in microbial iron acquisition (29). To investigate whether *DhSip1* influences fungal adhesive knob development and pathogenicity by regulating the subsequent iron acquisition process, we performed iron rescue experiments. Following the concentration screening (Fig. S3), the iron rescue experiment confirmed that supplementation with 10 μM FeSO_4_ (the minimal fully effective concentration) was sufficient to completely restore both the adhesive knob formation ability and the pathogenicity of the Δ*DhSip1* to the WT level (Fig. 5C through F). In contrast, the WT showed no significant change in either phenotype upon iron supplementation. To ascertain whether the rescue effect was specifically due to iron availability rather than the sulfate moiety, we conducted a parallel rescue experiment using an equivalent concentration (10 μM) of FeCl_3_ as an alternative iron source. The results demonstrated that FeCl_3_ supplementation also fully restored both the adhesive knob formation ability and the pathogenicity of the Δ*DhSip1* to the WT level (Fig. S4), confirming that the rescue effect in Δ*DhSip1* is mediated by the iron ion itself.

Collectively, our results support a model in which *DhSip1*, a key siderophore biosynthetic gene, is required for iron homeostasis. Its deletion impairs siderophore synthesis, resulting in cellular iron deprivation. This iron deficiency, in turn, impairs the expression of trap morphogenesis-related genes and inhibits adhesive knob formation, ultimately reducing the predatory efficacy of *D. haptotyla*.

### Subcellular localization and expression dynamics of *DhSip1*

To elucidate the role of *DhSip1* in the pathogenic process, we generated a fluorescent protein-tagged strain in *DhSip1* OE background to monitor its cellular localization and dynamics during nematode infection. It should be noted that as the endogenous expression signal in the WT was likely too weak for direct visualization, conducting this analysis in *DhSip1* OE effectively revealed its potential localization pattern.

**Fig 6:**
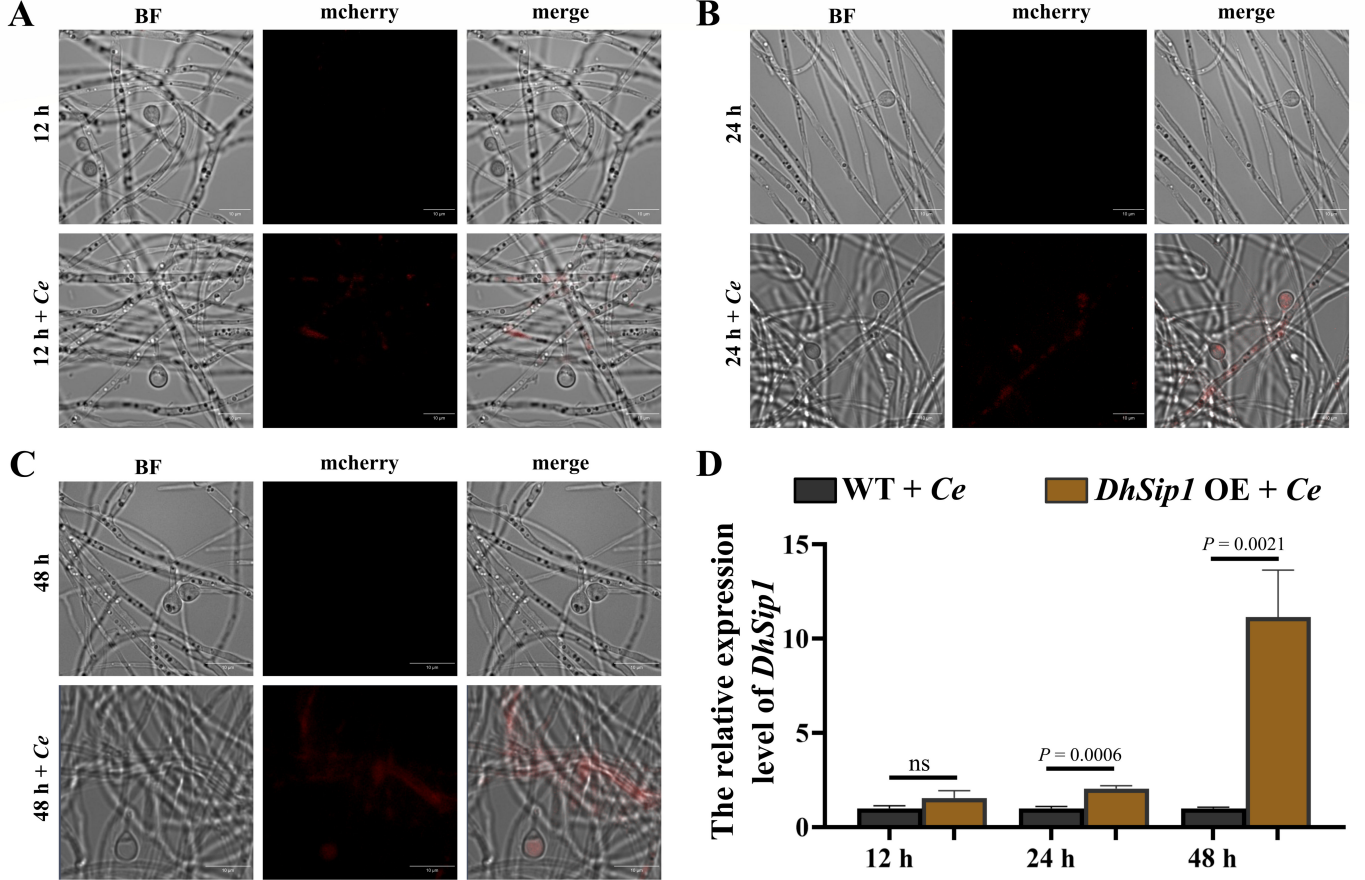
Expression intensity and localization of *DhSip1*. (**A–C**) Expression visualization of *DhSip1* in *DhSip1* OE under non-predation (12, 24, and 48 h; *DhSip1* OE strain only) and predation (12 h + *Ce*, 24 h + *Ce*, and 48 h + *Ce*). *Ce*, *C. elegans*. (**D**) Expression of *DhSip1* in the WT and *DhSip1* OE after induction by *C. elegans*. Values are means ± SD. ns represents *P* values >0.05; ** represents *P* values <0.01, which is calculated by unpaired Student’s *t*-test (two tailed); *n* = 3 biological replicates.

The results showed that the fluorescence signal of *DhSip1* exhibited strict nematode-dependent induction: a clear signal was detected only during interaction with nematodes but not in the basic medium without nematodes (Fig. 6A through C). More importantly, its expression displayed a distinct spatiotemporal pattern. At early stages of infection (12 h), the signal was primarily localized to the vegetative hyphae (Fig. 6A). As the infection progressed to the later stage (24 and 48 h), the fluorescence was abundantly detected in both the vegetative hyphae and adhesive knobs (Fig. 6B and C). Consistently, RT-qPCR results confirmed that the transcriptional level of *DhSip1* during infection was higher in the *DhSip1* OE than that in the WT (*P* < 0.01 at 24 and 48 h, unpaired Student’s *t*-test, *n* = 3; Fig. 6D).

Collectively, these findings indicate that *DhSip1* is a nematode-inducible gene, and its product accumulates in the adhesive knobs during the late predation. This specific spatial localization pattern, coupled with the observed phenotype of extensive knob formation in the *DhSip1* OE, suggests that the enrichment of its product in adhesive knobs constitutes an important cytological basis for its role in regulating predatory efficiency.

## DISCUSSION

The biosynthesis of siderophores and the subsequent iron acquisition processes are crucial for the virulence of pathogenic microorganisms (13, 30). This study provides many lines of experimental evidence demonstrating the important role of the NIS-type siderophore biosynthetic gene *DhSip1* in the nematode-trapping fungus *D. haptotyla*.

Fungi have evolved complex iron uptake systems to adapt to iron-limiting conditions, primarily involving the secretion of siderophores to chelate extracellular ferric iron and the utilization of cell surface reductive iron assimilation (RIA) systems (31, 32). The contribution of siderophore metabolism to pathogenicity has been established in various fungi (33, 34). For example, in *A. fumigatus*, disruption of siderophore biosynthetic genes significantly attenuates its virulence in both mice and fly models (35). These precedents reveal the universal importance of iron metabolism in fungal pathogenesis.

In this study, we first characterized the function of *DhSip1* in the predation process of *D. haptotyla* through genetic approaches. The concomitant severe reduction in trap (adhesive knob) formation and nematode mortality observed in Δ*DhSip1*, coupled with the opposite phenotypes in *DhSip1* OE, collectively identify the critical role of *DhSip1* in predation. Furthermore, our iron rescue assay provided direct causal evidence: exogenous iron supplementation completely restored both adhesive knob formation and nematode mortality in Δ*DhSip1*. This finding definitively anchors *DhSip1* function within the iron acquisition pathway, demonstrating that its deletion impairs siderophore synthesis, leading to insufficient iron uptake that fails to sustain adhesive knob morphogenesis, ultimately resulting in reduced pathogenicity. However, the deletion of *DhSip1* does not affect fungal vegetative growth. We propose that this discrepancy stems from the differential iron demands in these two conditions: trace amounts of iron are sufficient for hyphal growth, whereas the pathogenic process requires a substantial iron supply to support adhesive knob development. Consequently, Δ*DhSip1* exhibits significantly reduced pathogenicity under conditions of exogenous iron limitation during infection. In addition, the stress response phenotypes of the *DhSip1* OE strain—increased sensitivity to SDS and NaCl coupled with enhanced resistance to H_2_O_2_—support that disturbing *DhSip1* expression disrupts cellular iron homeostasis. We propose that iron overload in the *DhSip1* OE strain creates a cellular state that is primed for damage by specific stresses. Elevated intracellular iron potentiates Fenton reaction-driven lipid peroxidation, compromising baseline membrane integrity and rendering cells hypersensitive to the membrane-disrupting agent SDS (36, 37). Concurrently, iron-induced mitochondrial dysfunction likely impairs ATP synthesis (38, 39), weakening the cell’s capacity to maintain ionic homeostasis under NaCl stress. In contrast, the same iron surplus may fortify iron-dependent antioxidant systems by providing an augmented pool of the essential iron cofactor (40), thereby conferring enhanced resistance to direct oxidative challenge by H_2_O_2_.

Notably, the restoration of pathogenicity in Δ*DhSip1* upon exogenous iron supplementation implies the existence of siderophore-independent iron acquisition pathways in *D. haptotyla*; the conserved RIA system, which relies on ferric reductases and high-affinity iron transporters for direct iron uptake, may compensate for the loss of siderophore-mediated iron acquisition in Δ*DhSip1*. In contrast, functional complementation by other siderophore synthesis genes is unlikely, as transcriptome data demonstrated that the only other predicted siderophore biosynthesis-related gene (*EVM06G002410*) was clearly downregulated during predation (Fig. S5) (26). The failure of the homologous gene to compensate for the loss of *DhSip1*, together with the complete phenotypic rescue by iron supplementation, underscores the specialized role of the *DhSip1*-dependent NIS in supporting adhesive knob development during nematode predation. Collectively, these findings highlight the functional redundancy of iron uptake systems in *D. haptotyla*, which ensures the flexibility of iron acquisition strategies under iron-deficient conditions and maintains the fitness of *D. haptotyla* in complex environments.

These findings also offer insights into how pathogenic fungi overcome host-imposed nutritional immunity. Nutritional immunity is a key defense strategy whereby animals and insects inhibit pathogen invasion by restricting the availability of essential nutrients such as iron (41, 42). To break through this limitation, pathogenic fungi have evolved high-affinity iron acquisition mechanisms, primarily mediated by siderophores, to enhance their survival under iron-restricted conditions (43, 44). For example, in several fungal pathogens, mutants defective in siderophore biosynthesis show severely attenuated virulence, underscoring the critical role of iron acquisition in fungal pathogenicity (45–47). Therefore, the siderophore synthesis and iron uptake mediated by *DhSip1* likely play a crucial role in enabling *D. haptotyla* to overcome nematode nutritional immunity.

Beyond iron signaling, other nutrient stresses are widely involved in regulating fungal morphogenesis (48). For example, carbon or nitrogen starvation differentially activates the expression of the *brlA*, regulating asexual sporulation in *Aspergillus fumigatus* (49). In NTF, nematode is thought to trigger nitrogen starvation and induce trap development through processes such as autophagy (50). These observations suggest a universal regulatory role for nutrient signaling in fungal development. On this basis, we further investigated the spatiotemporal coupling between siderophore synthesis and trap development. Fluorescence localization analysis revealed that the expression of *DhSip1* is strictly nematode inducible, and its product accumulated in adhesive knobs during the late stages of infection. This observation indicates that the role of *DhSip1* is not global but precisely localized to the predation process. We speculate that this localization strategy establishes a relatively iron-enriched microenvironment within the adhesive knob, thereby providing immediate and efficient iron support for the maintenance and functionality of these energy-demanding structures.

In conclusion, our work not only confirms the critical function of *DhSip1* in siderophore biosynthesis and iron acquisition (essential for pathogenicity) but also, more significantly, elucidates its role in linking cellular iron nutrient homeostasis to adhesive knob development, thus optimizing the predatory efficiency of *D. haptotyla*. However, the precise molecular mechanisms through which *DhSip1*-mediated iron acquisition facilitates the formation of adhesive knobs remain an intriguing question to be elucidated. We propose several hypotheses worthy of future investigation: (i) as an essential cofactor for the mitochondrial electron transport chain and numerous metabolic enzymes, iron may support the generation of sufficient ATP and biosynthetic precursors for this energy-demanding developmental process; and (ii) iron might influence specific morphogenetic signaling pathways, such as the MAPK or cAMP-PKA cascades, by modulating ROS homeostasis or acting as a signaling molecule itself. Elucidating these specific mechanisms will provide deeper insights into the pathogenesis mediated by *DhSip1*.

## MATERIALS AND METHODS

### Strains and culture conditions

The WT strain of *D. haptotyla* was maintained at the State Key Laboratory for Conservation and Utilization of Bio-Resources in Yunnan province (Yunnan university) and cultured at 28°C on PDA medium. The mutant strains (Δ*DhSip1* and *DhSip1* OE) were cultured on PDA medium with 100 μg/mL hygromycin B. *Escherichia coli* DH5α, which was used for routine cloning, was cultured at 37°C on Luria-Bertani medium. *Caenorhabditis elegans* was routinely cultured at 25°C on nematode growth medium with *E. coli* OP50 as the food source. PDA, TG, and TYGA were used for the phenotypic analysis, as previously described (27).

### Bioinformatics analysis of *DhSip1* and phylogenetic tree construction

The annotation of the 3.2 gene cluster was performed by antiSMASH fungal version 7.0.0 (https://fungismash.secondarymetabolites.org/). The sequences used for phylogenetic analysis were downloaded from NCBI. MEGA 12 software was used in the construction of phylogenetic tree. The theoretical isoelectric point and molecular weight of *DhSip1* were predicted using the Compute pI/Mw tool on the ExPASy server.

### Generation of *DhSip1* deletion and overexpression strains

Ex taq DNA polymerase (TaKaRa, Kusatsu, Japan) was used for PCR. Restriction enzymes (New England Biolabs) and In-Fusion HD ligase (Clontech) were used, respectively, for the digestion and ligation of DNA fragments.

The deletion and overexpression were performed by means of PEG-mediated homologous recombination (27). For the deletion plasmid construction, around 2 kb of the 5′ and 3′ DNA sequences of *DhSip1* was amplified by PCR using *D. haptotyla* genomic DNA as the template. These homologous DNA fragments contained overlapping regions with neighboring fragments. Hygromycin resistance gene cassette (*hyg*) was amplified from pCSN44. The above three fragments (5′ flank, 3′ flank, and *hyg*) were assembled into the linearized pRS426 (digested with *Xba*I and *Sal*I) using the In-Fusion HD Cloning Kit (Clontech) to generate the *DhSip1* deletion vector pYUZ1001. For the overexpression plasmid construction, an additional promoter *gpda* was amplified from pYUZ87, and fragments (5′ flank, 3′ flank, *gpda*, *hyg*, and *DhSip1*) were assembled into the linearized pRS426 (digested with *Kpn*I and *Bam*HI) using the In-Fusion HD Cloning Kit (Clontech) to generate the *DhSip1* overexpression vector pYUZ1002. pYUZ1001 and pYUZ1002 were used as templates to obtain the linear *DhSip1* deletion and overexpression cassette for protoplast transformation. Protoplast preparation and gene targeting were carried out after partial optimization of the previously described methods (51). Transformants were selected on PDA containing 100 µg/mL hygromycin B and further confirmed by PCR (Fig. S1). Primers used in this study are provided in Table S1.

### Total RNA extraction, RT, and qPCR analysis

The WT and mutant strains (Δ*DhSip1* and *DhSip1* OE) were cultured in TG medium at 28°C for 3 days with shaking at 180 rpm to harvest tiny mycelia. Then the mycelia were filtered, collected, and added into 1% sucrose solution at 28°C for 2–3 days. The Δ*DhSip1* and *DhSip1* OE interaction groups (Δ*DhSip1* + Ce and *DhSip1* OE + Ce) were established by adding approximately 300–400 *C. elegans* to a 1% sucrose solution. Fungal-nematode interaction samples were collected at 12, 24, and 48 h post-induction for both groups. WTs under identical treatment conditions were labeled as CD12, CD24, and CD48; WTs under identical treatment conditions but without nematodes were used as control groups and labeled as D12, D24, and D48.

Total RNA was extracted from each of the fungal samples using the RNA Extraction Kit (Axygen, Suzhou, China); 2 μg pure RNA was used for reverse transcription with the FastQuant RT Kit (Takara). qPCR was performed with SYBR Premix Ex Taq II kit (Takara) in the Roche LightCycler 480 System (Roche Applied Science) under standard running conditions, except that the last step was done at 50°C. The β-tubulin of *D. haptotyla* (EVM07G006950) was used as the internal control for normalization. The fold change of *DhSip1* was calculated using the 2^−ΔΔCT^ method. The primers used in this study are provided in Table S2.

### Comparison of vegetative hyphae growth

WT and mutant strains (Δ*DhSip1* and *DhSip1* OE) were cultured on PDA medium for 10 days until fresh mycelial spread over the plate. Fresh fungal disks (7 mm diameter) of WT and mutant strains were then inoculated on TG, TYGA, and PDA media at 28°C for 8 days. The growth of colonies was recorded, and the diameter of the colonies was measured daily.

### Chemical stressor response assay

TG medium supplemented with different concentrations of chemical stressors, including osmotic stressors NaCl (0.1, 0.2, and 0.3 mol/L), oxidative stressor H_2_O_2_ (5, 10, and 15 mM), cell wall stress agent sodium dodecyl sulfate (SDS) (0.01%, 0.02%, and 0.03%), and Congo red (0.03, 0.06, and 0.09 mg/mL), was used for the stress response test. TG medium without chemical stressors was used as the control. WT and the mutant strains (Δ*DhSip1* and *DhSip1* OE) were inoculated onto the above media at 28°C for 8 days. The growth diameter of the colonies was measured daily. The stress tolerance was assessed with colony growth rates, as previously described (52).

### Pathogenicity assay

The WT and mutant strains (Δ*DhSip1* and *DhSip1* OE) were first cultured in TG medium at 28°C with shaking (180 rpm) for 3 days to generate tiny mycelia. The mycelia from each strain were then collected, and equal amounts were spread onto the WA plates (3 cm). All plates were incubated at 28°C for 3 days. Prior to the pathogenicity assay, the mycelial growth and density were confirmed to be similar between the WT and mutant strains. Subsequently, 150–200 *C. elegans* were added to each plate. After 12, 24, and 48 h of the *C. elegans* addition, the number of adhesive knobs and the nematode mortality rates were recorded. The entire assay was conducted in triplicate.

### Siderophore detection and iron rescue assay

#### CAS assay

To verify siderophore production, the CAS assay was performed as described previously (53). Briefly, the CAS solution was poured into the plates, then fungal colonies were inoculated on the CAS plates. Fungal capacity to produce siderophore was indicated by the pale-yellow zone around the colony.

#### Iron rescue assay

WT and mutant strains were first cultured in TG medium for 2 days. The hyphae were then evenly spread on the WA plates (containing 10 µM FeSO_4_), and 150–300 individuals of *C. elegans* were added. After 12, 24, and 48 h of the *C. elegans* addition, the number of adhesive knobs and the nematode mortality rates were recorded.

### Fluorescence microscopy

mCherry was amplified from the pCAMBIA1300-mCherry and with overlapping regions. The mCherry was assembled into the linearized pYUZ1002 (digested with *Kpn*I and *Bam*HI) using the In-Fusion HD Cloning Kit (Clontech) to generate the *DhSip1* fluorescence location vector pYUZ1003. The subcellular localization of *DhSip1* was observed in the *DhSip1* OE strain. Hyphae were pre-cultured in TG medium for 2 days and transferred to 1% sucrose for 3 days, and then 150–300 individuals of *C. elegans* were added. Cocultures were maintained at 28°C in darkness and sampled at 12, 24, and 48 h. Hyphae of the *DhSip1* OE strain without the *C. elegans* were used as the control. Hyphal bodies and adhesive knobs were analyzed using a super-resolution microscope (Nikon SIM).

### Statistical analysis

All tests were carried out with three biological replicates. GraphPad Prism 8.0 (San Diego, CA, USA) was used for data analysis. Data are presented as mean ± SD. Statistical analyses were performed using either an unpaired two-tailed Student’s *t*-test or a two-way ANOVA followed by Tukey’s multiple comparison test. In all cases, a *P* value of less than 0.05 was considered statistically significant.

## SUPPLEMENTAL MATERIAL

Supplemental material aem.02446-25-s0001.docx Fig. S1 to S5; Tables S1 and S2.

ASM does not own the copyrights to Supplemental Material that may be linked to, or accessed through, an article. The authors have granted ASM a non-exclusive, world-wide license to publish the Supplemental Material files. Please contact the corresponding author directly for reuse.
