# Supplementary material for: DhSip1, an NRPS-independent siderophore biosynthetic gene, regulates adhesive knob formation and pathogenicity in Dactylellina haptotyla through mediating iron acquisition
Source: Appl Environ Microbiol. 2026 Mar 30;92(4):e02446-25. doi: 10.1128/aem.02446-25 (PMC13101506; doi:10.1128/aem.02446-25)
Supplement: Supplemental material — Fig. S1 to S5; Tables S1 and S2. [file aem.02446-25-s0001.docx]

*DhSip1*, an NRPS-independent-siderophore biosynthetic gene, regulates adhesive knob formation and pathogenicity in *Dactylellina haptotyla* through mediating iron acquisition

Hong-Mei Lei ^a, b,^ ^&^, Shi-Mei Shen ^a,^ ^&^, Ping Xu ^a^, Guo-Hong Li ^a^, Pei-Ji Zhao ^a, *^

^a^ State key Laboratory for Conservation and Utilization of Bio-Resources in Yunnan, School of Life Sciences, Yunnan University, Kunming, 650500, China.

^b^ School of Ecology and Environmental Sciences, Yunnan University, Kunming, 650500, China.

^&^ These authors contributed equally to this work.

* Correspondence should be sent to [pjzhao@ynu.edu.cn](mailto:pjzhao@ynu.edu.cn).


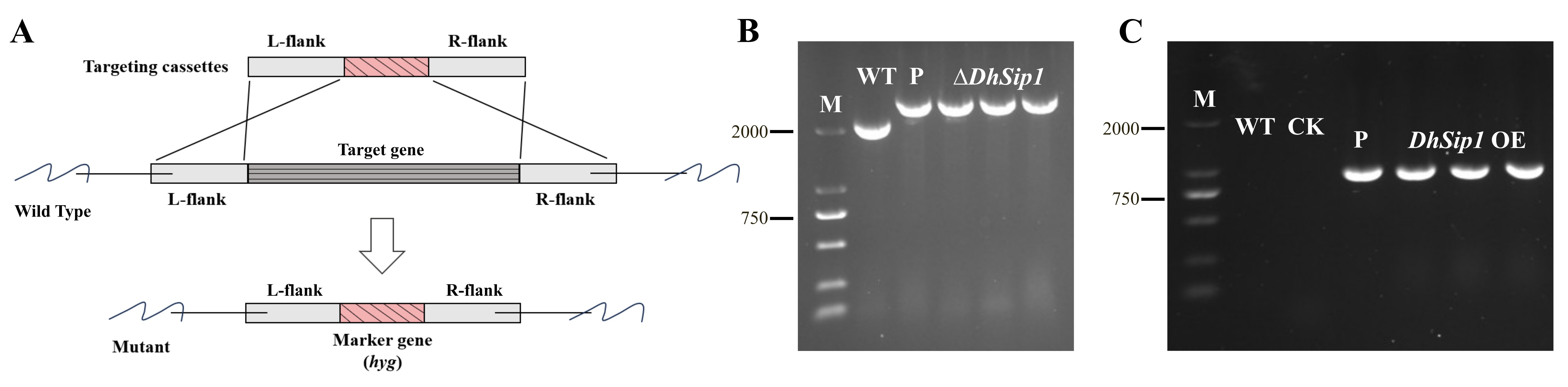


**Fig. S1** *DhSip1* deletion and overexpression in *Dactylellina haptotyla* YMF1.03409. (A) Diagrammatic sketch of homologous recombination. (B) PCR verification of Δ*DhSip1*. (C) PCR verification of *DhSip1* OE.


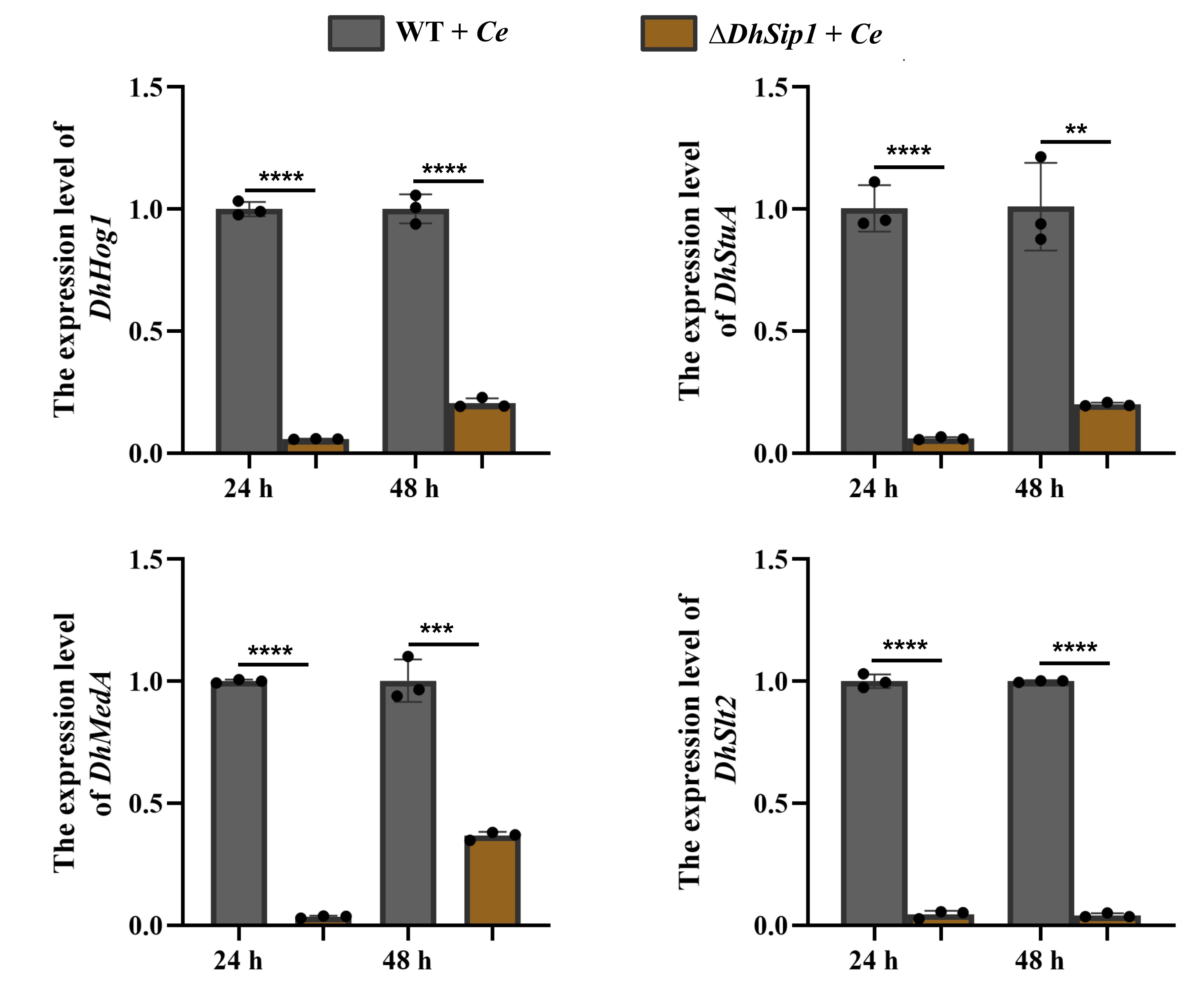


**Fig. S2** The relative expression level of trap-development related genes in Δ*DhSip1* strain predation on *C. elegans*.

**
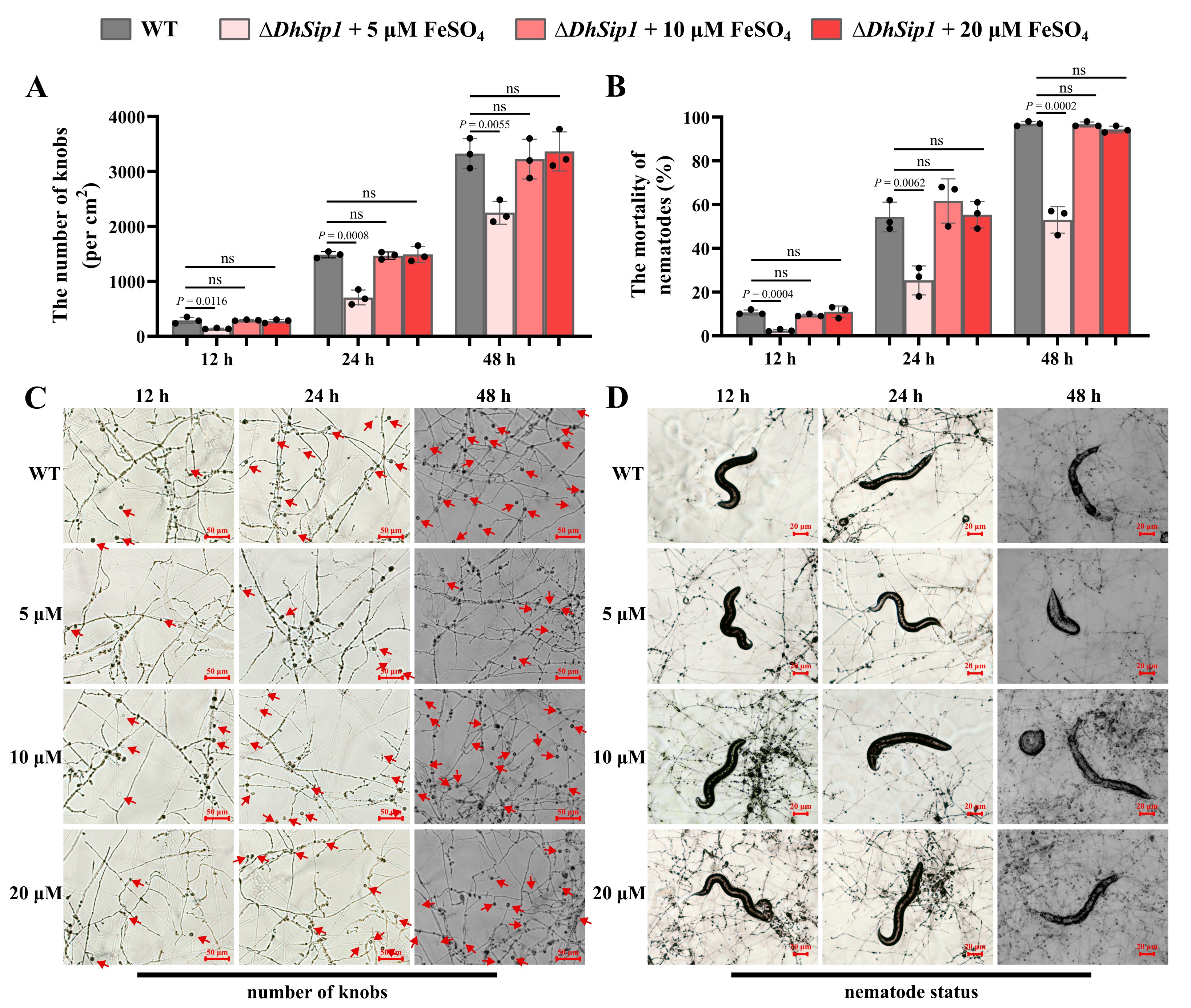
**

**Fig. S3** Selection of the optimal FeSO_4_ concentration for iron rescue assays. (A) Comparison of adhesive knob number between the Δ*DhSip1* (treated with gradient concentrations of FeSO_4_) and the untreated WT. (B) Comparison of nematode mortality between the Δ*DhSip1* (treated with gradient concentrations of FeSO_4_) and the untreated WT. (C) and (D) Representative views of Δ*DhSip1* (treated with gradient concentrations of FeSO_4_) and the untreated WT.

**
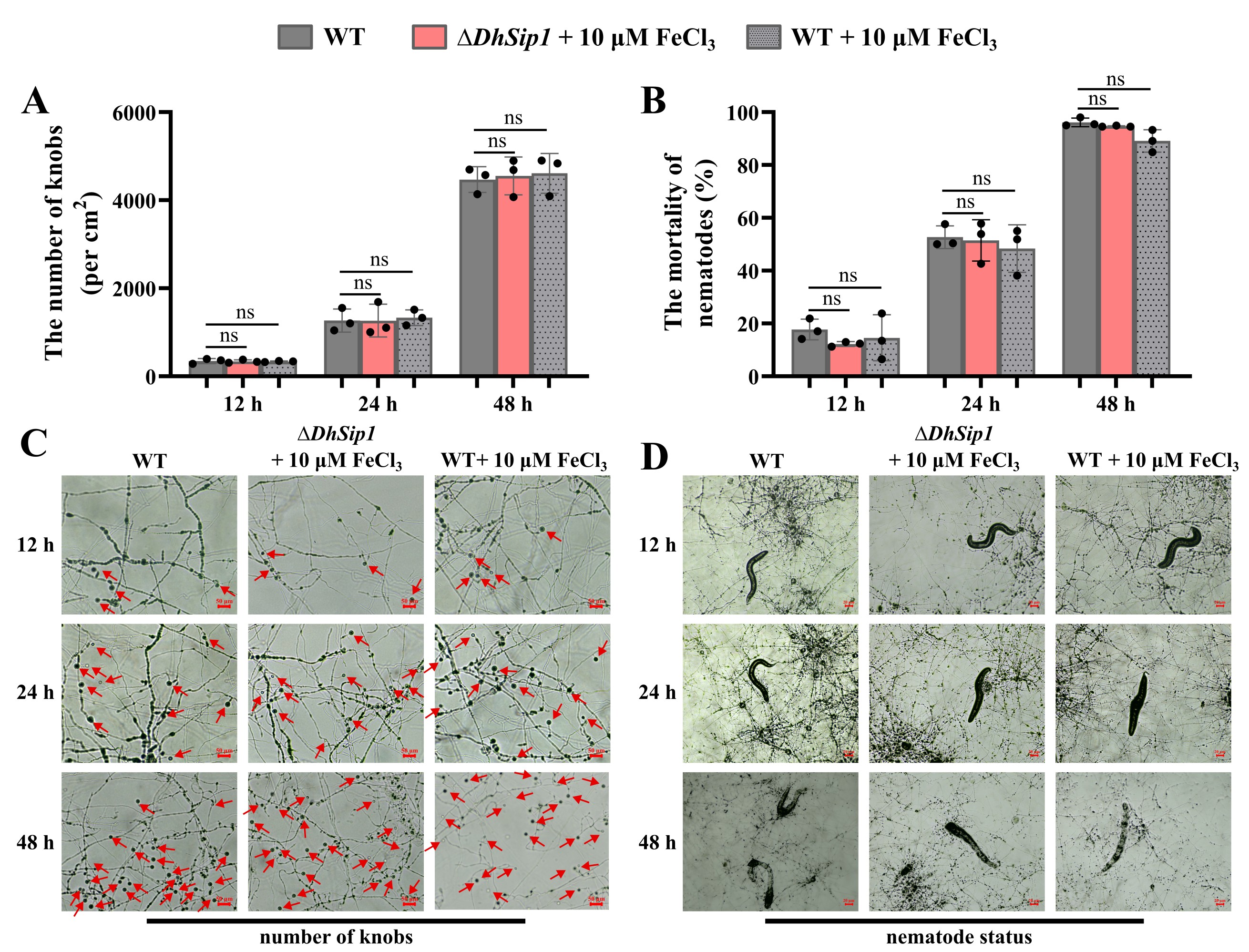
**

**Fig. S4** Iron rescue assay using 10 μM FeCl_3_ as an alternative iron source. (A) and (B) Iron rescue assay and its effect on pathogenicity. (C) and (D) Representative views of WT, Δ*DhSip1+*FeCl_3_, and WT*+*FeCl_3_ control at different time points after interaction with *C. elegans*.

**
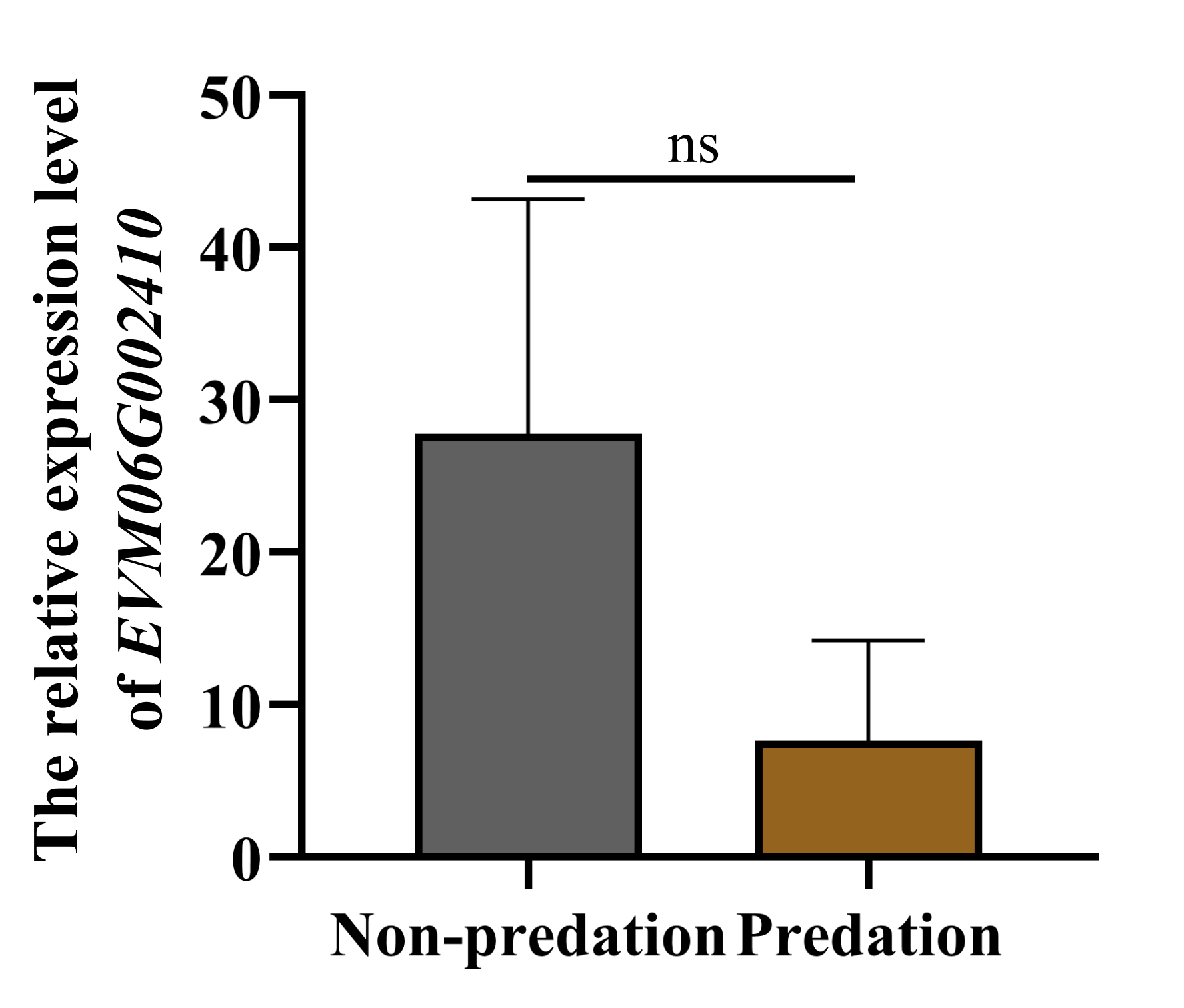
**

**Fig. S5** Transcript levels of the predicted siderophore biosynthesis-related gene *EVM06G002410* in *D. haptotyla* under nematode predation and non-predation conditions.

**Table S1.** Primers for gene disruption and overexpression used in this study.

| **Name** | **Sequence** | **Length (bp)** |
| --- | --- | --- |
| *DhSip1*-ko-5f | ttcggatcttccagaCGGATTCTATTTTTTATTTTATTTTTTTC | 1547 |
| *DhSip1*-ko-5r | ccttcaatatcatcttctgTAAAGGTTATTAGACTTTCATTCGCG |  |
| *DhSip1*-ko-3f | tccttctttACGGGTCTGTCGTTCAACGA | 1571 |
| *DhSip1*-ko-3r | caactgccgttcgacTCTAGCTCCTGTTGCAAGATCCC |  |
| *Hyg*-ko-f | aCAGAAGATGATATTGAAGGAGCATTT | 2133 |
| *Hyg*-ko-r | gacagacccgtAAAGAAGGATTACCTCTAAACAAGTGTACC |  |
| ko-ver-f | CTGTTGGTGTTGGATTTTTTTG | 2788 |
| ko-ver-r | CATTTTAGTGCCATCGTGTCGC |  |
| *DhSip1*-oe-5f | ACCGAAATATAAAACCCTTC | 922 |
| *DhSip1*-oe-5r | GACAATTAGCCTCTCAATCA |  |
| *DhSip1*-oe-3f | CAAGCAATGCAGACTAAATGCG | 910 |
| *DhSip1*-oe-3r | TGTGGTGACAAGATGTCCCAAG |  |
| oe-ver-f | CTGTTGGTGTTGGATTTTTTTG | 910 |
| oe-ver-r | CATTTTAGTGCCATCGTGTCGC |  |

**Table S2.** RT-qPCR primers used in this study.

| **Name** | **Sequence (5'-3')** | **Length (bp)** |
| --- | --- | --- |
| *β*-tubulin-f | GATGGCTCCGGTGTCTA | 211 |
| *β*-tubulin-r | CAGTTGTTACCGGCGC |  |
| *DhStuA*-f | CCGCTCCAAACTCACAGACC | 108 |
| *DhStuA*-r | CGACCAAAATTGCGAATCCC |  |
| *DhSlt2*-f | GTGCGCCTGAGATCATGTTGA | 165 |
| *DhSlt2*-r | CATTGGGGGTACCGAGGTAGT |  |
| *DhMedA*-f | CAGCCTCCAACGACTTGACTT | 175 |
| *DhMedA*-r | GGAACCTTCTCGCCGTACCAT |  |
| *DhHog1*-f | ATCCAAGACCCCCAGATGACC | 151 |
| *DhHog1*-r | GGAACAACGGCTTTCCCTCCA |  |
| *DhSid1*-f | AAAGAGCAGGGAGTGAAGAAAA | 158 |
| *DhSid1*-r | GCCAACGAAGCTGTAGAGAATT |  |
